# Crucial role of androgen receptor in resistance and endurance trainings-induced muscle hypertrophy through IGF-1/IGF-1R- PI3K/Akt- mTOR pathway

**DOI:** 10.1186/s12986-020-00446-y

**Published:** 2020-03-30

**Authors:** Lijun YIN, Lin LU, Xiaojing LIN, Xiaohui WANG

**Affiliations:** Department of Kinesiology, Shanghai University of Sport, 188 Hengren Road, Yangpu District, Shanghai, 200438 People’s Republic of China

**Keywords:** Androgen receptor, Muscle hypertrophy, Training, IGF-1, IGF-1R, PI3K/Akt/mTOR

## Abstract

**Background:**

Androgen receptor (AR) has been reported to play vital roles in exercise-induced increase of muscle mass in rats, but needs to be further verified and the mechanism behind remains unclear. As AR target genes, insulin growth factor-1 (IGF-1) and IGF-1 receptor (IGF-1R) promote muscle hypertrophy through activating PI3K/Akt- mammalian target of rapamycin (mTOR) pathway, a classic pathway of muscle hypertrophy. So the main purpose of this study was using AR antagonist flutamide to demonstrate AR’s effect on training-induced muscle hypertrophy and its possible mechanism: IGF-1/IGF-1R- PI3K/Akt- mTOR pathway?

**Methods:**

Forty-eight Sprague Dawley male rats aged 7 weeks were randomly divided into six groups: control (C), flutamide (F), resistance training (R), resistance training plus flutamide (R + F), endurance training (E), and endurance training plus flutamide (E + F) groups. Flutamide was used to block AR in rats. Rats in R and R + F groups fulfilled 3 weeks of ladder climbing with progressively increased load, while E and E + F rats completed 3-week moderate intensity aerobic exercise on a treadmill. The relative muscle mass (muscle mass/body weight) of rats was detected. Serum levels of testosterone and IGF-1 of rats were determined by ELISA, and mRNA levels of IGF-1R and mTOR in muscles by real-time PCR. Protein levels of AR, IGF-1, IGF-1R, mTOR, PI3K, Akt, p-PI3K and p-Akt in muscles were detected by Western blot.

**Results:**

(1) The training-induced rise in the relative muscle mass and the expression levels of AR were only found in the gastrocnemius of R rats and in the soleus of E rats (selective muscle hypertrophy), which were blocked by flutamide. (2) Serum testosterone in the R and E rat were increased, and flutamide exerted no effect. (3) The levels of IGF-1, IGF-1R and mTOR as well as the activities of PI3K and Akt were enhanced selectively (in the gastrocnemius of R rats and in the soleus of E rats), which were reduced by flutamide. **Conclusions:** AR exerted an essential role in both resistance training and endurance training-induced muscle hypertrophy, which was mediated at least partly through IGF-1/IGF-1R- PI3K/Akt- mTOR pathway.

## Introduction

Testosterone is widely reported to affect muscle mass and exercise capacity, with high level testosterone promoting, while low level declining muscle mass and exercise capacity. Exogenous androgen administration significantly facilitated the protein synthesis, prevented muscle proteolysis and increased the muscle mass and strength via the mediation of androgen receptor (AR) [1]. Due to the severe side-effects (cardiovascular events and benign prostatic hyperplasia) resulted from long-term or large dose of androgen administration, androgen supplementation is strictly limited [2], and selective androgen receptor modulators (SARMs) especially the non-steroidal tissue-specific SARMs were used currently to activate AR in specific tissue. Sufficient evidences demonstrated the enhancement of lean mass and strength obtained by SARMs in dystrophy muscle of female elderly with sarcopenia [3] and mice with myotonic dystrophy [4]. On the contrary, global AR gene knockout (ARKO) and specific skeletal muscle tissue ARKO mice showed alterations in the muscle mass (such as gastrocnemius, quadriceps and soleus) [5, 6], muscle glycogen [7] and exercise performance. The important role of AR in the increases of muscle mass and exercise performance at untrained state was demonstrated [5, 8–11], but whether AR exerted crucial role in exercise-induced muscle hypertrophy in vivo have not been thoroughly verified. To our best knowledge, until now no ARKO model mice have been used to explore AR’s role in exercise-induced changes in muscle mass and performance, moreover, ARKO mice could not be obtained commercially. Thus, AR specific antagonist flutamide is still widely employed to clarify AR’s role [12, 13].

The molecular mechanisms by which AR regulates exercise-induced alterations in muscle mass remain largely unknown. It is widely accepted that insulin-like growth factor 1 (IGF-1) plays vital roles in the proliferation of myoblasts and muscle hypertrophy. Previous studies have observed the significant increase of muscle mass in mice with over-expression of IGF-1 and also revealed the decrease of IGF-1 in mice with attenuated muscle mass caused by malnutrition, diseases or aging. The biological functions of IGF-1 are mediated by IGF-1R. Moreover, AR’s effects on facilitating untrained muscle hypertrophy are at least partly associated with IGF-1/IGF-1R, reflected as androgen supplementation greatly increased the muscle size accompanied with an increase of IGF-1 expression [14], while AR blockage significantly blunted the testosterone-induced myotube formation and enlarged diameters, and reduced the mRNA level of IGF-1R in C2C12 myoblast [15].

IGF-1/IGF-1R contributes to muscle hypertrophy on untrained state mainly through activating phosphatidylinositol 3 kinase (PI3K)/protein kinase B (Akt)- mammalian target of rapamycin (mTOR) pathway [16–18], evidenced as IGF-1 induced-growth promotion of skeletal muscle could be greatly inhibited by PI3K inhibitor LY294002 or Akt inhibitor KP372–1 [19]. PI3K/Akt- mTOR pathway is activated by phosphorylating PI3K, Akt and mTOR successively after PI3K translocation to phosphorylated insulin receptor substrate 1 [18], then exerted its vital roles in regulating muscle development and hypertrophy by promoting proliferation and muscle protein synthesis and preventing muscle degradation as well [20]. Tight relationship of AR with PI3K/Akt- mTOR pathway has been revealed by considerable amount of evidences, for example, PI3K/Akt inhibitor significantly reduced testosterone-induced muscle hypertrophy, and in turn, AR antagonist flutamide offset the increase of muscle mass caused by androgen supplementation accompanied with the decreased levels of IGF-1R and p-Akt [21, 22], which indicated that androgen/AR-induced muscle hypertrophy in untrained participants was at least partly associated with the activation of IGF-1- Akt/mTOR pathway. But whether the effects of AR on muscle hypertrophy at trained state was mediated by IGF-1- Akt/mTOR pathway remains unproved.

Our previous studies using mechanical stretch of myoblasts to mimic muscle movement have revealed an indispensable role of AR and its mechanism in vitro -- through the mediation of IGF-1/IGF-1R- PI3K-mTOR pathway, according to two facts: (1) mechanical stretch-induced pro-proliferation of C2C12 myoblasts (highly expressed AR) was reversed by flutamide, or IGF-1R blockade or PI3K/Akt inhibition [23]; (2) stretch-induced pro-proliferation was greatly attenuated in L6 myoblasts (without AR expression) compared to C2C12 myoblasts, and the proliferation of L6 myoblast was promoted by either AR over-expression or IGF-1 supplement, then inducing the activation of IGF-1R- PI3K- mTOR pathway [24].

Therefore, in the current study, flutamide was used to block AR signaling in male rats to confirm the positive effect of AR on exercise-induced muscle hypertrophy in vivo and further explore its mechanisms: IGF-1/IGF-1R- PI3K/Akt- mTOR pathway?

## Materials and methods

### Animals and grouping

Forty-eight Sprague-Dawley (SD) male rats aged 7 weeks (170–190 g body weight) were purchased from Beijing Vital River laboratory Animal Technology Co. Ltd., and housed under standard specific pathogen free (SPF) conditions in a temperature and humility-controlled room on a 12-h light:12-h dark cycle. Food and water were freely available throughout the study. The animal protocol was approved and all the experimental procedures were supervised by the Ethics Committee of Shanghai University of Sport (No. 2018002). After acclimating to laboratory conditions for 3 days, rats were randomly and evenly divided into six groups: control (C), AR antagonist flutamide embedded (F), resistance training (R), resistance training plus flutamide (R + F), endurance exercise (E) and endurance exercise plus flutamide (E + F).

### Flutamide treatment

Rats in F group, E + F group and R + F group were anesthetized and embedded with flutamide releasing pellet (50 mg/pellet, evenly releases for 21 days, Innovative Research of America, USA.) prior to exercise intervention. Briefly, after anesthesia, an almost 0.5 cm long incision was cut on the neck, and pellet was implanted into the incision site, then gently sutured the incision. Rats in C group also underwent the surgery but no pellet embedded.

### Exercise protocol

**Fig. 1:**
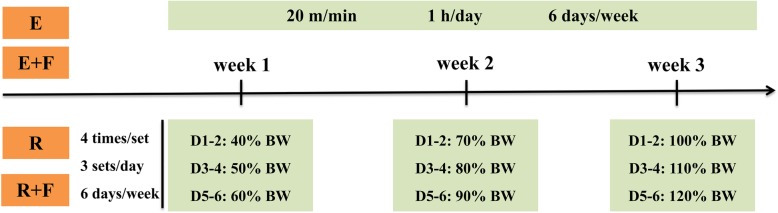
Exercise protocol. Rats in E and E + F groups participated in a moderate intensity aerobic exercise on a treadmill at a speed of 20 m/min for one hour each time, while the rats in R and R + F groups underwent resistance training with progressively incremental load. E: endurance training; E + F: endurance training plus flutamide embedded; R: resistance training; R + F: resistance training plus flutamide embedded; BW: body weight; D1–2 means day 1 and day 2

As shown in Fig. 1, after one-day adaptation to endurance or resistance training, rats in E and E + F groups participated in a moderate intensity aerobic exercise on a treadmill at a speed of 20 m/min for 1 hour each time, while the rats in R and R + F groups underwent resistance training, which comprises three sets of ladder climbing with progressively incremental load. Briefly, rats start climbing at the bottom of the ladder (1 m in height, 2 cm distance from each stairs and a slope of 85°) with progressively increased load on tail (the initial weight attached to tail was 40% of body weight, and increased by 10% every 2 days until reached 120% of body weight). Every set consists of four repetitions and three sets (20 s interval period between sets) in each time. Resistance or endurance training lasts for 3 weeks and 6 days per week.

### Detection of serum testosterone and IGF-1 by ELISA

Serum testosterone and IGF-1 levels of rats were measured by ELISA according to manufacturer’s instructions. The intra- and inter-assay coefficients of variation (CV) were less than 2.9 and 6.8% for detecting testosterone (KGE010, R&D systems, Minnesota Minneapolis, USA) as well as 4.1 and 4.3% for IGF-1 kit (MG100, R&D systems, Minnesota Minneapolis, USA).

### Quantitative real-time PCR

Rats were anaesthetized at 36 h after the last exercise, gastrocnemius and soleus were collected and frozen in liquid nitrogen then stored at − 80 °C until analyzed or immediately processed as the following process. Total RNA was extracted using Trizol reagent (Invitrogen, CA, USA), the first strand cDNA was synthesized by Revert Aid First Stand cDNA Synthesis Kit (Thermo Scientific, MA, USA) in accordance with the manufacturer’s instructions. Primers for amplification genes of IGF-1R (sense: 5′-AAA CGC TGA CCT CTG TTA CCT-3′, antisense: 5′-ACG CCT TTG TAG TAG TAG TGT CG-3′), mTOR (sense: 5′-GAT ACG CCG TCA TTC CTC-3′, antisense: 5′-TGC TCA AAC ACC TCC ACC-3′) and GAPDH (sense: 5′-GCT GAG TAT GTC GTG GAG-3′, antisense: 5′-TCT TCT GAG TGG CAG TGA T-3′) were synthesized by Sangon Biotech Co, Ltd. Shanghai. 50 ng of cDNA templates were added into FastStart universal SYBR Green Master (Roche company, Switzerland) to amplify the genes above, and the amplification conditions for these genes were the same: 10 min denaturation at 95 °C followed by 40 cycles of 15 s denaturation at 95 °C, 60 s annealing and elongation at 60 °C. The mRNA values of IGF-1R and mTOR of samples were corrected by that of the internal control of GAPDH and shown as the ratios of target genes to GAPDH.

### Western blot

About 50 mg of gastrocnemius and soleus were cut into pieces and homogenized after adding 500 μL of radioimmunoprecipitation assay (RIPA) buffer containing phenylmethanesulfonyl fluoride (PMSF) (Beyotime Biotechnology, China) (RIPA:PMSF = 100:1) to extract total protein. After sonication on the ice, lysates were centrifuged at 14000 rpm for 20 min at 4 °C. Then supernatants were collected and protein concentration was determined by BCA protein assay kit (Beyotime Biotechnology, Shanghai, China). Extracts (50 μg) of gastrocnemius or soleus was fractionated on 10% SDS-PAGE gels, then electro-transferred onto nitrocellulose membranes. After blocking with 5% nonfat milk for 2 h, the nitrocellulose membranes were incubated overnight at 4 °C with primary antibodies against AR and IGF-1R (both 1:500, Santa Cruz Biotechnology, CA, USA), PI3K, Akt, mTOR, p-PI3K and p-Akt (all 1:1000, Cell Signaling Technology, MA, USA). After washing three times with Tris-buffered saline with 0.1% Tween 20 (TBST), each time for 5 min, the bands were incubated with horseradish peroxidase (HRP) conjugated secondary antibodies (Santa Cruz Biotechnology, CA, USA) for 1 h at room temperature. Then, the bands were washed again as described above, developed with Immbilon Western chemiluminescent HRP substrate (Millipore, MA, USA), and visualized by automatic chemiluminescence image analysis system (Tanon Biotechnology, Shanghai, China). The density of blot was determined using Bio-image software (Tanon Biotechnology, Shanghai, China) and normalized against GAPDH.

### Statistical analysis

Statistical analysis of data was performed using *SPSS* for Windows 19.0 software package (IBM Corporation, Armonk, NY, USA). All data were presented as Mean ± SD. Statistical difference for C, F, R and R + F groups, as well as the C, F, E and E + F groups were determined by one-way analysis of variance (ANOVA) and post hoc comparison using least significant difference (LSD)-t test. The level of statistical significance was set as *p* < 0.05.

## Results

### Flutamide reversed the training-induced selective muscle hypertrophy

**Fig. 2:**
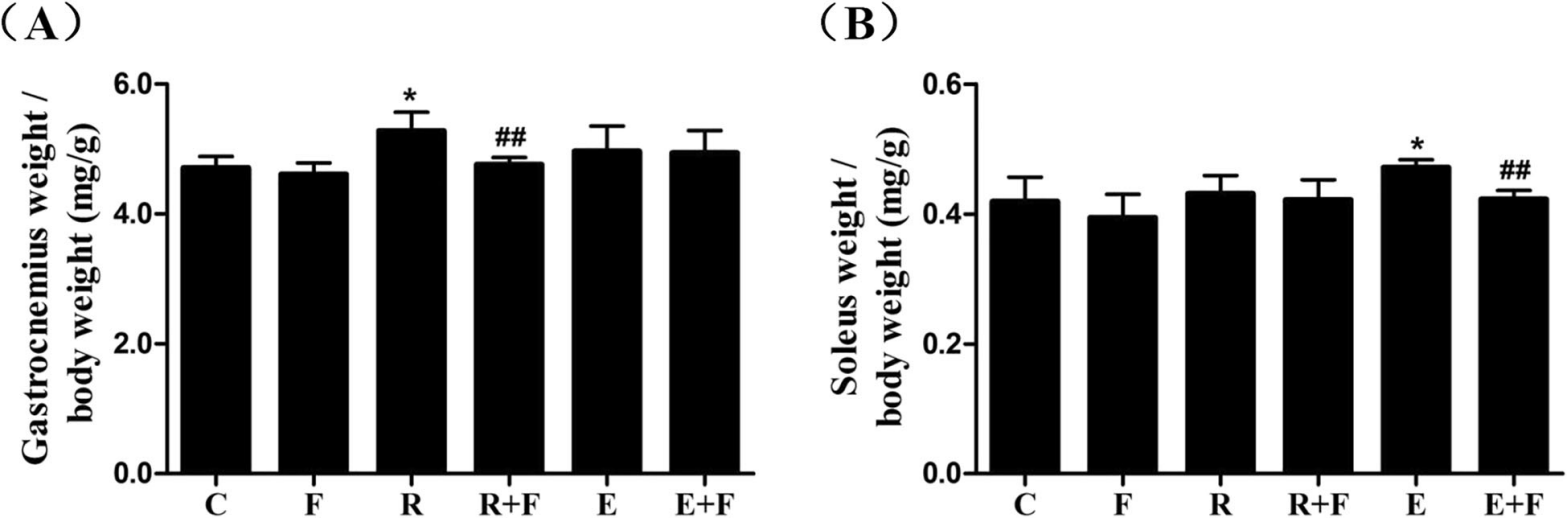
Flutamide reversed the training-induced selective muscle hypertrophy. Resistance training and endurance training increased the relative muscle mass of gastrocnemius (**a**) and soleus (**b**) respectively, which was reversed by flutamide treatment. The ratio of muscle weight with body weight is considered as the relative muscle mass. C: control; F: flutamide embedded; R: resistance training; R + F: resistance plus flutamide embedded; E: endurance training; E + F: endurance plus flutamide embedded. ^*^P<0.05 vs C; ^##^*P*<0.01 vs the relative training group

Compared with control, the relative muscle mass of gastrocnemius but not soleus was increased in R rats (Fig. 2a), while enhanced muscle mass was observed only in the soleus of E rats (Fig. 2b), indicating a selective muscle hypertrophy induced by trainings (means resistance training mainly results in hypertrophy of fast switch muscle such as gastrocnemius, while endurance training mainly induces hypertrophy of slow switch muscle such as soleus). Furthermore, flutamide could reverse the training-induced selective muscle hypertrophy (Fig. 2).

### Flutamide reversed the training-induced selective increase of AR protein level in muscles instead of promotion of testosterone in serum

**Fig. 3:**
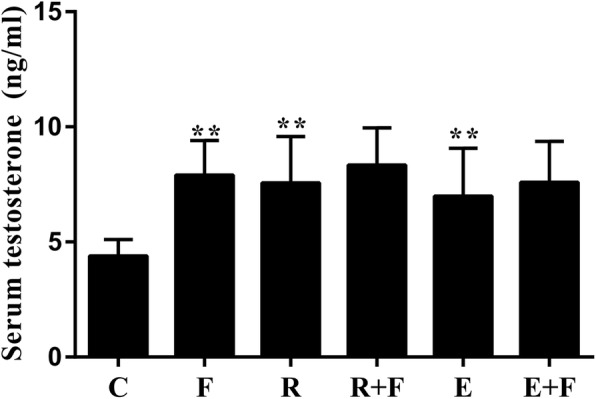
Effects of trainings and flutamide on the serum testosterone. Both resistance training and endurance training increased the serum testosterone concentration. Flutamide did not alter the level of serum testosterone in either resistance training or endurance training rats although an increase of serum testosterone was found in sedentary rats. The levels of serum testosterone in rats were detected by ELISA. C: control; F: flutamide embedded; R: resistance training; R + F: resistance training plus flutamide embedded; E: endurance training; E + F: endurance training plus flutamide embedded. ^**^P<0.01 vs C

The serum levels of testosterone in R and E rats were significantly increased compared to control, and blocked AR by flutamide did not change the levels of serum testosterone in the training rats, while flutamide elevated serum testosterone of the untrained control rats (Fig. 3).

**Fig. 4:**
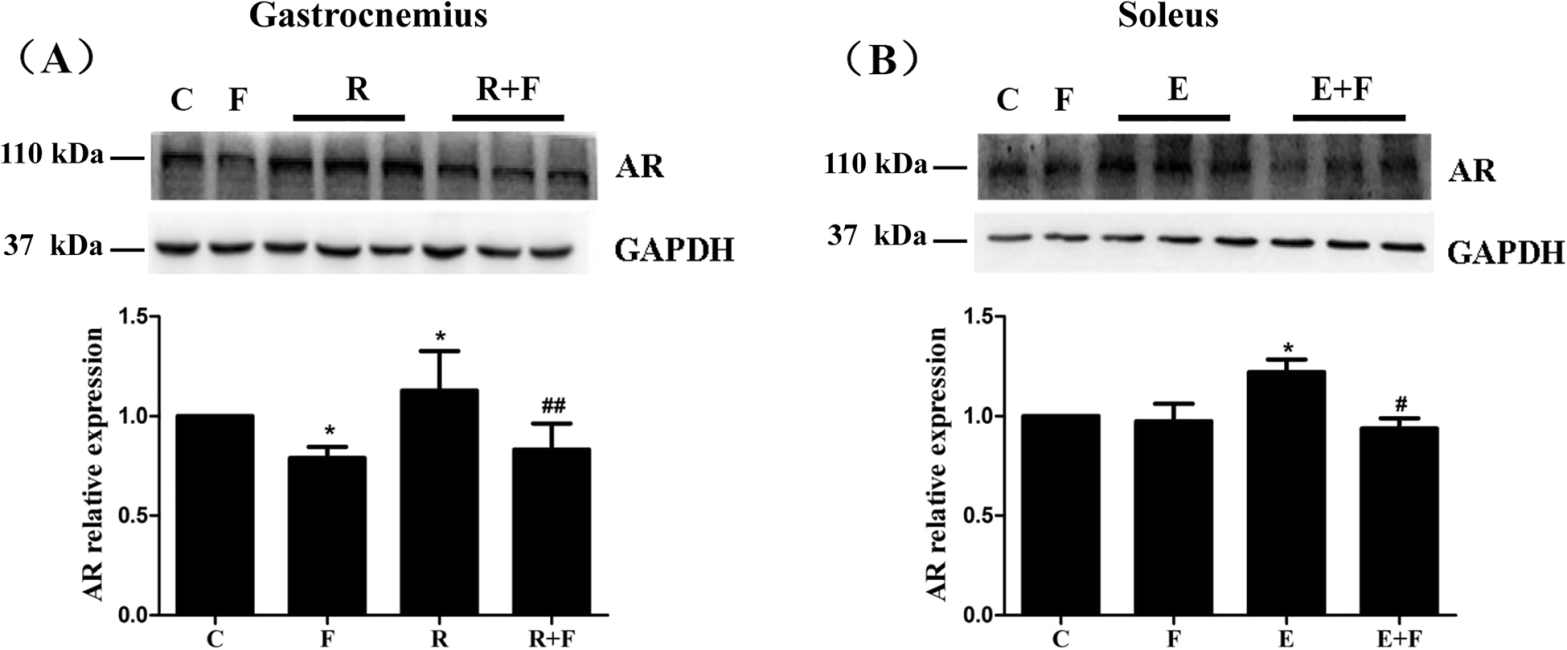
Flutamide reversed the training-induced increases of AR in the muscles of rats. The elevated protein levels of AR in the gastrocnemius through resistance training (**a**) and in the soleus through endurance training (**b**) were both reversed by flutamide treatment. Protein levels of AR and GAPDH in gastrocnemius and soleus were determined by Western blot, and levels of AR were quantified and normalized against GAPDH. The AR/GAPDH ratio of control group was identified as 1, and the relative values of AR in experimental groups are expressed as fold of control. C: control; F: flutamide embedded; R: resistance training; R + F: resistance training plus flutamide embedded; E: endurance training; E + F: endurance training plus flutamide embedded. Data are expressed as the mean ± SD of at least three independent experiments. ^*^P<0.05 vs C; ^#^*P*<0.05, ^##^*P*<0.01 vs the relative training groups

Accompanied with the training-induced selective muscle hypertrophy, AR also showed a selective increase, characteristic of up-regulated AR only in gastrocnemius of R rats (Fig. 4a) and in soleus of E rats (Fig. 4b) compared with control rats, while no alteration of AR was observed in non-hypertrophic muscles including the soleus of R rats and the gastrocnemius of E rats (data not shown). Furthermore, the selective increases of AR in the hypertrophy muscles of R and E rats were noticeably blocked by flutamide, and an attenuation of AR in gastrocnemius but not in soleus of F rats was found compared with control (Fig. 4).

### Flutamide lowered serum IGF-1 of the trained rats

**Fig. 5:**
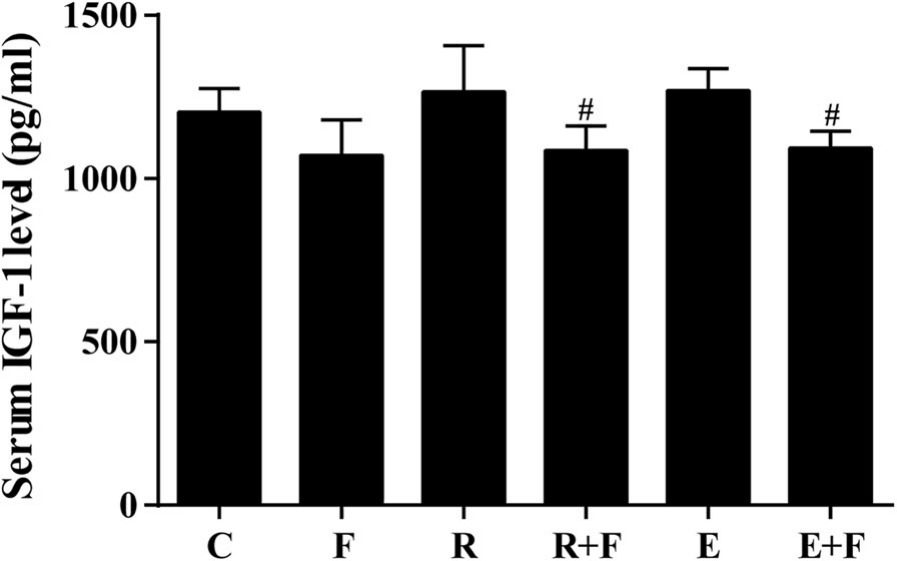
Flutamide lowered the serum IGF-1 concentration of training rats. Flutamide decreased the serum concentration of IGF-1 in both resistance training and endurance training rats although no change was found in rats with flutamide treatment or training alone. C: control; F: flutamide embedded; R: resistance training; R + F: resistance training plus flutamide embedded; E: endurance training; E + F: endurance training plus flutamide embedded. ^#^*P*<0.05 vs the relative training groups

As shown in Fig. 5, obvious attenuation of serum IGF-1 concentration was found in R + F rats compared with R rats and in E + F rats compared with E rats, although no noticeable alteration of IGF-1 was induced by resistance or endurance training.

### Flutamide reversed the training-induced selective enhancement of IGF-1R and mTOR

As shown in Table 1, significant increases of the mRNA levels of IGF-1R and mTOR were observed in gastrocnemius of R rats or in soleus of E rats compared with control rats, and moreover, the two trainings-induced increases in IGF-1R and mTOR were reversed by flutamide.

**Table 1:** Flutamide reversed the enhancement of IGF-1R and mTOR at mRNA levels induced by trainings

	*n*	Gastrocnemius		Soleus	
		IGF-1R	mTOR	IGF-1R	mTOR
C	7	1.00 ± 0.23	1.00 ± 0.18	1.00 ± 0.20	1.00 ± 0.21
F	6	0.88 ± 0.11	0.91 ± 0.11	0.81 ± 0.14	0.85 ± 0.14
R	8	1.32 ± 0.21^*^	1.43 ± 0.10^**^	/	/
R + F	8	0.96 ± 0.06^##^	0.97 ± 0.16^##^	/	/
E	8	/	/	1.30 ± 0.20^*^	1.32 ± 0.28^*^
E + F	7	/	/	0.98 ± 0.18^#^	0.94 ± 0.27^##^

Note: The increased mRNA levels of IGF-1R and mTOR were found in the gastrocnemius of resistance training rats and in the soleus of endurance training rats. Flutamide reversed the trainings-induced changes in IGF-1R and mTOR at mRNA level. *C* Control, *F* Flutamide embedded, *R* Resistance training, *R + F* Resistance training plus flutamide embedded, *E* Endurance training, *E + F* Endurance training plus flutamide embedded. ^*^P<0.05, ^**^P<0.01 vs C; ^#^*P*<0.05, ^##^*P*<0.01 vs the relative training groups

**Fig. 6:**
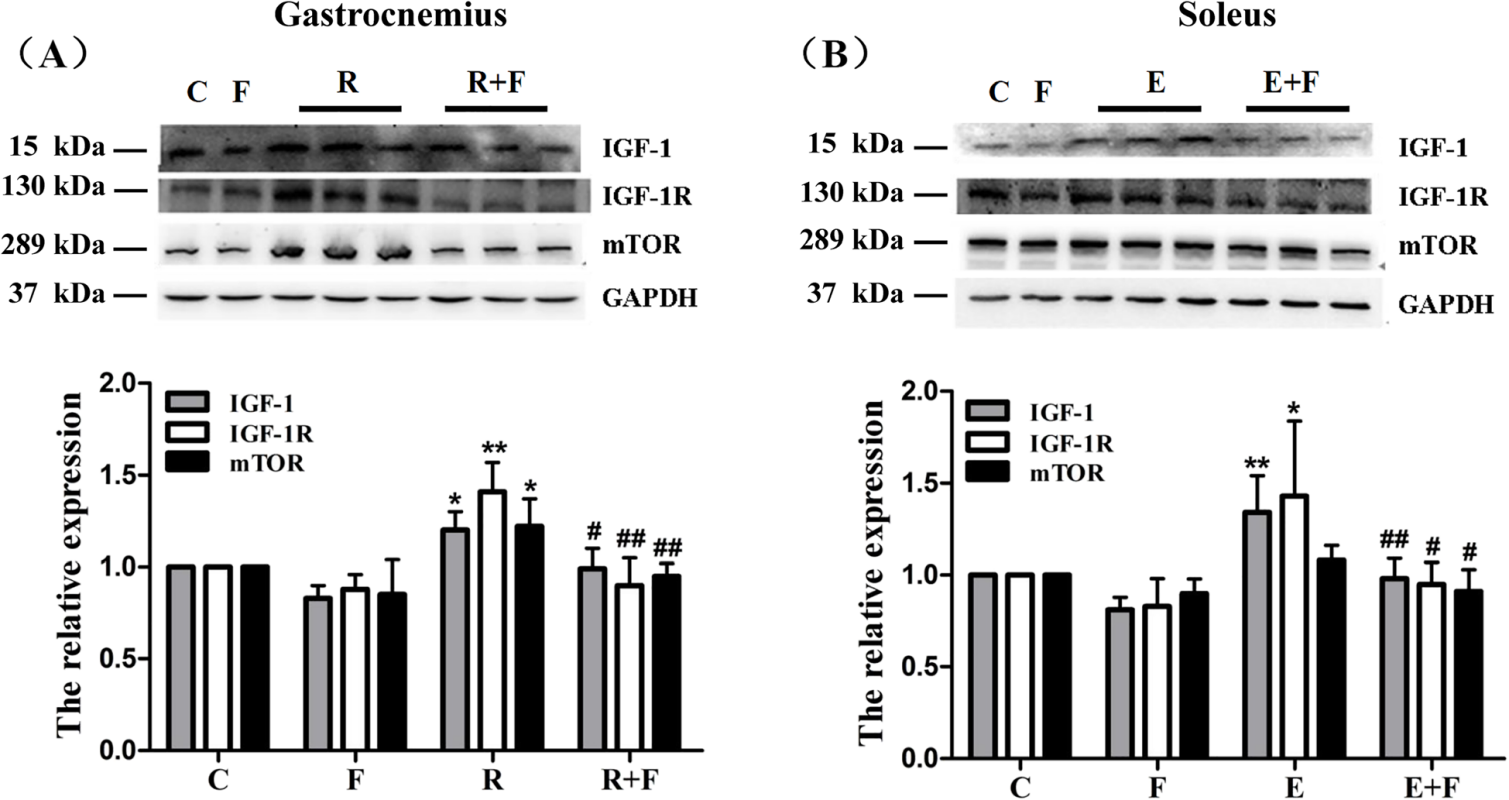
Flutamide down-regulated the protein levels of IGF-1, IGF-1R and mTOR in the muscles of training rats. **a**: in gastrocnemius, resistance training-induced elevations in the protein levels of IGF-1, IGF-1R and mTOR were reversed by flutamide treatment; **b**: in soleus, endurance training raised the protein levels of IGF-1 and IGF-1R rather than mTOR, but all of them were decreased by flutamide treatment. Protein levels of IGF-1, IGF-1R, mTOR and GAPDH in gastrocnemius and soleus were determined by Western blot, and levels of IGF-1, IGF-1R, and mTOR were quantified and normalized against GAPDH. The ratio of IGF-1/GAPDH, IGF-1R/GAPDH, or mTOR/GAPDH in control group was identified as 1, and the relative values of the above indicators in experimental groups are expressed as fold of control. C: control; F: flutamide embedded; R: resistance training; R + F: resistance training plus flutamide embedded; E: endurance training; E + F: endurance training plus flutamide embedded. Data are expressed as the mean ± SD of at least three independent experiments. ^*^P<0.05, ^**^P<0.01 vs C; ^#^*P*<0.05, ^##^*P*<0.01 vs the relative training groups

Similar results were obtained on the protein levels of IGF-1, IGF-1R and mTOR. Compared with control rats, the protein levels of IGF-1, IGF-1R and mTOR were enhanced only in gastrocnemius of R rats or soleus of E rats (but no change of mTOR in E rats) (Fig. 6), and no alterations of IGF-1, IGF-1R and mTOR were found in non-hypertrophic muscles including the soleus of R rats and the gastrocnemius of E rats (data not shown), indicated selective increases of IGF-1, IGF-1R and mTOR at protein levels in the hypertrophy muscle by the two trainings. Furthermore, the protein levels of IGF-1, IGF-1R and mTOR were down-regulated in R + F rats compared with R rats and in E + F rats compared with E rats (Fig. 6).

### Flutamide reversed the training-induced selective increases in expressions and activities of PI3K and Akt

**Fig. 7:**
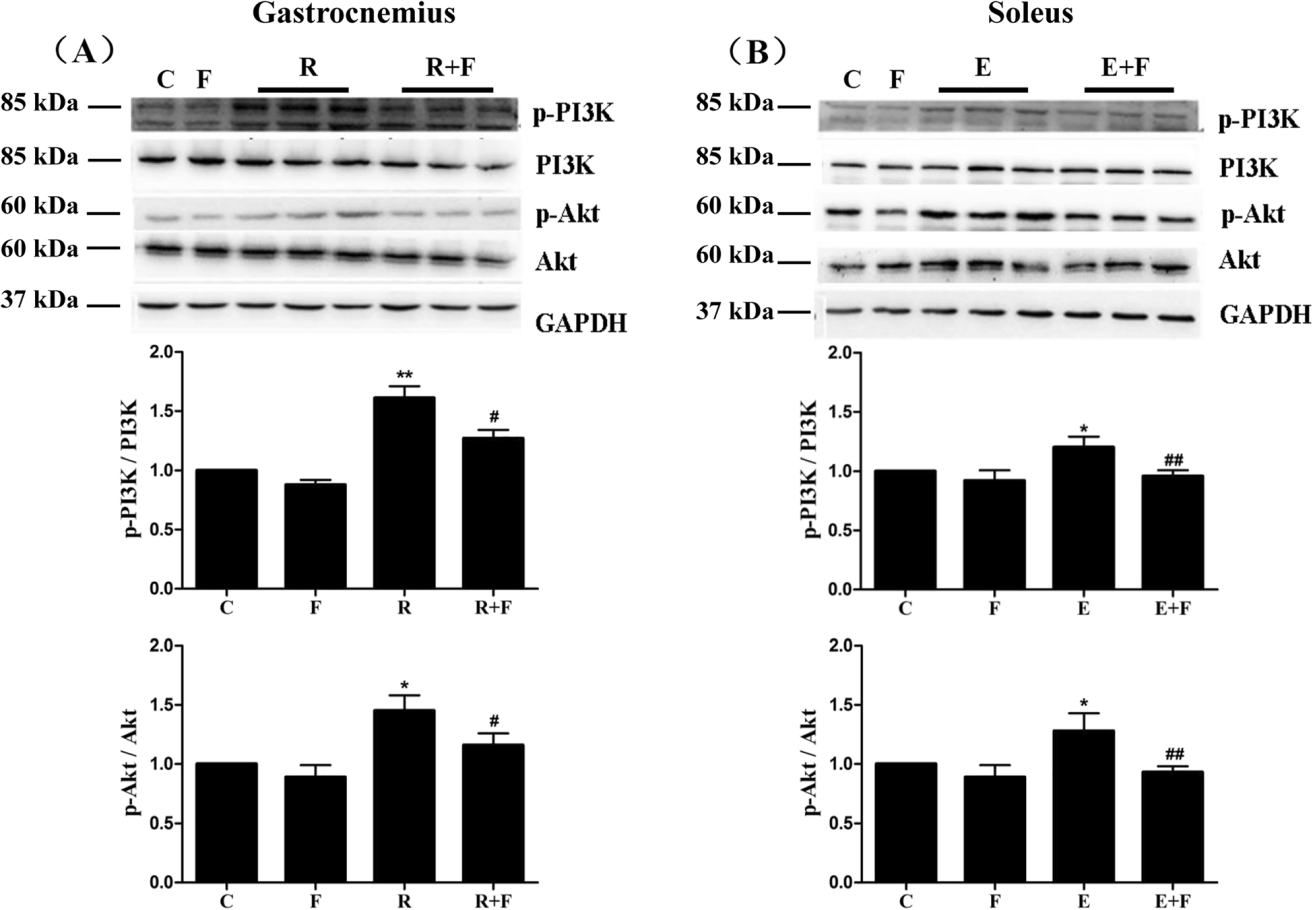
Trainings induced the enhancements of PI3K and Akt in activities rather than protein levels in the muscles of rats, which were reversed by flutamide. Elevations of p-PI3K/PI3K ratio and p-Akt/Akt ratio in the gastrocnemius through resistance training (**a**) and in the soleus through endurance training (**b**) were reversed by flutamide treatment. No change was observed in the ratios of PI3K/GAPDH and Akt/GAPDH among groups (so the graphs do not show). Protein levels of p-PI3K, PI3K, p-Akt, Akt and GAPDH in gastrocnemius and soleus were determined by Western blot, and protein levels and activity of PI3K were reflected by PI3K/GAPDH ratio and p-PI3K/PI3K ratio, respectively. Akt’s protein level and activity were described similarly. The p-PI3K/PI3K ratio or p-Akt/Akt ratio of control group was identified as 1, and the relative values of p-PI3K/PI3K or p-Akt/Akt in experimental groups are expressed as the fold of control. C: control; F: flutamide embedded; R: resistance training; R + F: resistance training plus flutamide embedded; E: endurance training; E + F: endurance training plus flutamide embedded. Data are expressed as the mean ± SD of at least three independent experiments. ^*^P<0.05, ^**^P<0.01 vs C; ^#^*P*<0.05, ^##^*P*<0.01 vs the relative training groups

Significant increases in activities rather than expression levels of PI3K and Akt were observed in the gastrocnemius of R rats (Fig. 7a) or in the soleus of E rats (Fig. 7b) compared with control rats, and no changes were observed in the activities and expression levels of PI3K and Akt in non-hypertrophic muscles including the soleus of R rats and the gastrocnemius of E rats (data not shown), indicated the training-induced selective increases of IGF-1, IGF-1R and mTOR at protein levels in the hypertrophy muscles. Furthermore, as shown in Fig. 7, the selective rises in the activities of PI3K and Akt resulted from the two trainings were decreased by flutamide. In addition, flutamide failed to change the activities of PI3K and Akt of untrained control rats.

## Discussion

### Crucial effects of AR in training-induced selective muscle hypertrophy

The effects of AR on boosting strength and endurance capacity through increasing muscle mass have been further verified in ARKO mice, reflected as a significant decrease of lean mass in multiple muscles including long extensor digitorum, tibialis anterior, gastrocnemius and soleus by 15%~ 22% in global ARKO mice [25] or in hypothalamus ARKO mice [26]. But most of the reports about AR’s role were focused on the sedentary participants. At training state, AR was likely to be very important not only for muscle hypertrophy but also strength promotion, for example, the submaximal running endurance and the maximum time to exhaustion were reduced in flutamide-treated rats [10] and in human, the intramuscular AR content influenced the cross-sectional areas of muscle in previously trained men [11]. Although muscle hypertrophy is a vital factor to enhance exercise performance, the roles of AR in exercise-induced muscle hypertrophy are not fully clarified, let alone the underlying molecular mechanisms. In this study, we found that endurance exercise induced the hypertrophy of soleus (typical slow-twitch muscle) while resistance training contributed to the hypertrophy of gastrocnemius (mostly made of type Π or fast-twitch fibers), that is training-induced selective muscle hypertrophy. Selective muscle hypertrophy resulted from trainings has been reporting for many years, for example, prolonged resistance training increased the cross-sectional area of type Π fiber in frail older adults but not in type Ι muscle fiber [27], and the abundant type Π muscle fibers are usually observed in elite weightlifters [28]. Moreover, in the present study, the training-induced selective muscle hypertrophy and selectively increased AR protein levels were observed, which were completely reversed by flutamide. Therefore, AR may play an indispensible role in both resistance training and endurance training-related muscle hypertrophy.

In addition, flutamide is considered to inhibit AR singling through blocking AR’s binding to its ligand like testosterone or dihydrotestosterone [29], and in the current study flutamide was found to reverse the training-induced increases of AR in muscles, suggested that flutamide might inhibit AR signaling through down-regulating the protein level of AR in tissue, except for the well-known blockade of binding with testosterone. Similar result was reported by a recent study which revealed the inhibitive function of flutamide by down-regulating AR protein in tissues [10].

### Mechanisms of AR’s role in training-induced muscle hypertrophy

AR affects muscle mass through genomic and non-genomic mechanisms. For genomic mechanism, AR mediates protein synthesis and degradation of skeletal muscle via modulating target genes such as myogenic regulatory factor (MRF) and ubiquitin ligases, which mostly cost more than 24 h to exert effects for completing transcription and translation of target genes. While the non-genomic mechanism of AR, characteristic of quick response to AR, recently have been proven an increasing importance in muscle protein balance (prompting protein synthesis and inhibiting degradation), is fulfilled through interactions of AR with other molecules such as IGF-1 and myostatin, especially IGF-1 [30].

The important positive effect of IGF-1 on muscle mass was demonstrated in humans including community-dwelling middle-aged and elderly adults [30], and IGF-1 is considered as a promising therapeutic agent for sarcopenia resulted from aging or hypogonadism and muscle weakness from staving [31]. As mentioned in the Introduction, AR’s role in increasing muscle mass at unstrained state was mediated by IGF-1/IGF-1R in vivo and in vitro [15], and our previous work in two kinds of myoblasts (C2C12 cells with high AR expression level, while L6 cells without detectable AR) using flutamide, AR over-expression, exogenous IGF-1 and IGF-1R neutralizing antibody (inhibiting IGF-1R signaling) demonstrated that the essential role of AR in myoblasts proliferation resulted from mechanical stretch (mimic muscle movement) was mediated by IGF-1/IGF-1R in vitro [23, 24]. This study further indicated that training-induced muscle hypertrophy in vivo was likely to be modulated through IGF-1/IGF-1R.

IGF-1/IGF-1R mainly exert their functions in facilitating proliferation of muscle cells and muscle hypertrophy on untrained or un-stretched state through stimulating PI3K/Akt and thereby activating mTOR [16, 32], and antagonist of PI3K could significantly inhibit the activation of mTOR in C2C12 cells [33]. For trained state, our previous work in vitro demonstrated the vital effects of IGF-1/IGF-1R on mechanical stretch-induced pro-proliferation of myoblasts by activating PI3K/Akt- mTOR using exogenous IGF-1, IGF-1R neutralizing antibody, and inhibitors of PI3K/Akt [23, 24]. In vivo, other researches’ evidences suggest the relation between IGF-1/IGF-1R and PI3K/Akt- mTOR because of the synchronous increase during exercise-promoted muscle hypertrophy [34, 35], but needs further verification. In the current study, accompanied with exercise-induced selective muscle hypertrophy, the protein levels of IGF-1, IGF-1R and mTOR and the activations of PI3K and Akt were increased only in the hypertrophy muscles but not in the non-hypertrophy muscle (selective enhancements in the levels and activities of the molecules mentioned above), suggested the roles of IGF-1/IGF-1R and PI3K/Akt- mTOR pathway. Furthermore, AR antagonist flutamide could reverse the exercise-induced increases in the protein levels of IGF-1, IGF-1R and mTOR and the activations of PI3K and Akt, accompanied with the reversed muscle hypertrophy. These results indicated that the crucial role of AR in training-induced muscle hypertrophy in vivo might be played through IGF-1/IGF-1R-PI3K/Akt-mTOR pathway. Evidence from other study supports our results, which demonstrated the synergistic effects of androgen/AR signaling on exercise-induced muscle hypertrophy via the activation of mTOR [36]. To our best knowledge, few reports about the relation between AR and PI3K/Akt- mTOR pathway in trained state in vivo has been found.

**Fig. 8:**
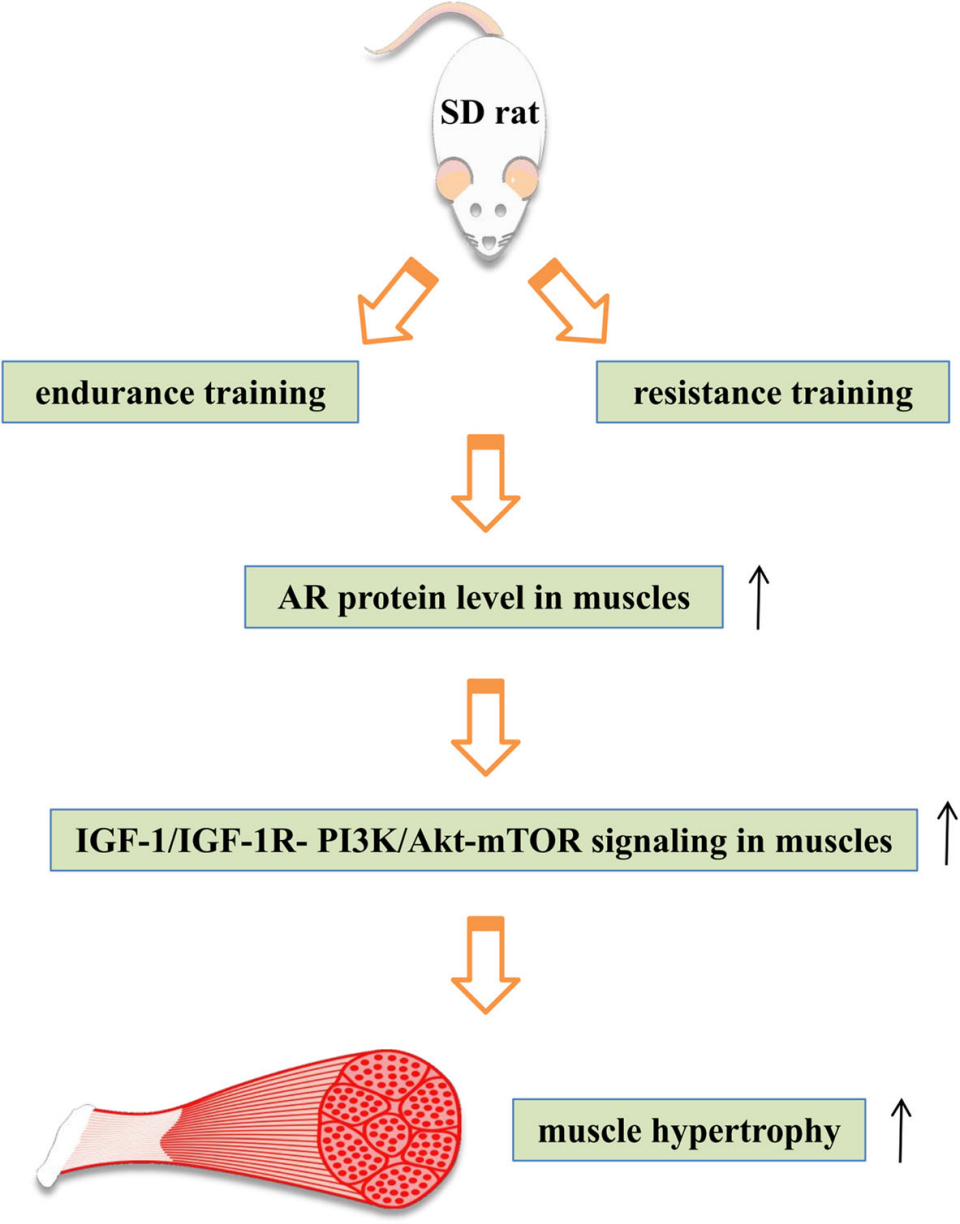
Summary diagram. Endurance and resistance trainings could raise AR protein levels in the muscle, then activating IGF-1/IGF-1R- PI3K/Akt- mTOR pathway in the muscle to promote muscle hypertrophy of male rats, which explained the reason in part of training-induced muscle hypertrophy. The thin upward arrowheads stand for increase or promotion

To sum up (Fig. 8), the present study firstly demonstrated a crucial effect of AR on either resistance or endurance training-induced muscle hypertrophy in vivo, and further suggested that AR’s role might be achieved through the mediation of IGF-1/IGF-1R-PI3K/Akt-mTOR pathway, which facilitates the understandings on the roles and mechanisms of AR in exercise-induced increases of muscle hypertrophy. However, a limitation that we have to consider in this study is that we have not demonstrated the indispensability of PI3K/Akt- mTOR pathway for AR’s roles in exercise-induced muscle hypertrophy, and inhibitors of PI3K, Akt and mTOR need to be used in rats in the future to clarify that question.

## Conclusions

AR played a crucial role in both resistance and endurance trainings-induced muscle hypertrophy of rats, which might be achieved at least partly through the mediation of IGF-1/IGF-1R- PI3K/Akt- mTOR pathway.

## Data Availability

The datasets analysed in this study are available from the corresponding author upon reasonable request.

## Abbreviations

AR: Androgen receptor
IGF-1: Insulin-like growth factor-1
mTOR: Mammalian target of rapamycin
PI3K: Phosphatidylinositol 3 kinase
Akt: Protein kinase B
ARKO: Androgen receptor gene knockout
MRF: Myogenic regulatory factor
